# Ways of understanding the regulation of physical activity among persons with knee osteoarthritis in digital rehabilitation: a phenomenographic study

**DOI:** 10.1136/bmjopen-2026-124247

**Published:** 2026-09-23

**Authors:** Johan Söderberg, Inger K Holmström, Maria Ehn, Ulrika Florin, Petra Von Heideken Wågert

**Affiliations:** 1 Department of Health Sciences, Innovation and Design, Mälardalen University, Västerås, Sweden; 2 Centre for Welfare Change, Mälardalen University, Västerås, Sweden; 3 School of Health, Care and Social Welfare, Mälardalens Universitet, Västerås, Sweden; 4 Public Health and Caring Sciences, Uppsala Universitet, Uppsala, Sweden; 5 Department of Computer Science and Engineering, Mälardalen University, Västerås, Sweden

**Keywords:** QUALITATIVE RESEARCH, eHealth, Exercise, Patient-Centered Care, Attitude

## Abstract

**Abstract:**

**Objectives:**

To explore how patients in digital rehabilitation for knee osteoarthritis understand the regulation of physical activity.

**Design:**

A qualitative, exploratory study with a phenomenographic approach.

**Setting:**

A digital rehabilitation programme for knee osteoarthritis in Sweden.

**Participants:**

20 adults with knee osteoarthritis were recruited using purposive sampling to maximise variation in sex, pain intensity, body mass index and fear avoidance.

**Results:**

Five qualitatively different ways of understanding the regulation of physical activity were identified, represented by: avoidance of physical activity, need for expert guidance, navigating symptoms and daily activity, adaptation to remain active and development through physical activity. The descriptive categories were related through shared dimensions concerning perceptions of physical activity, interpretation of pain, purpose of regulation and perception of the body. The categories differed in aspects considered important when regulating physical activity and in the range of actions perceived as possible.

**Conclusions:**

Different ways of understanding the regulation of physical activity shape the range of possible actions among patients in digital rehabilitation for knee osteoarthritis. Some ways of understanding appear to constrain regulatory options, whereas others support a wider range of strategies to remain physically active. These findings contribute to the understanding of why similar health information and recommendations may be interpreted differently and could lead to varying approaches to physical activity.

**Trial registration number:**

https://doi.org/10.17605/OSF.IO/N3UBQ.

**STRENGTHS AND LIMITATIONS OF THIS STUDY**

- The rich variation captured through in-depth interviews enabled the identification of distinct ways of understanding the regulation of physical activity.
- The phenomenographic approach allowed analysis of structural relationships across categories, supported by several dimensions of variation.
- Investigator triangulation within the interdisciplinary research team strengthened the analysis and reduced the risk of interpretive researcher bias through the inclusion of multiple disciplinary perspectives.
- The voluntary participation in a digital rehabilitation programme may have introduced selection bias, as participants may have been more motivated or more positive towards exercise than the broader osteoarthritis population, and the findings should therefore be interpreted within this specific context.
- Interviews were conducted through digital meetings, which may have limited the ability to observe non-verbal cues, although it also increased accessibility and may have facilitated reflection in a familiar environment.

## Introduction

 Osteoarthritis (OA) of the knee is the long-term condition that most frequently causes mobility disability in older adults, with rising prevalence leading to a substantial social and individual burden.^1 2^ The condition is associated with pain, functional limitations and reduced quality of life, and increases the risk of lifestyle-related diseases and all-cause mortality.^3 4^ OA is widely understood as a result of structural joint damage, where wear and tear leads to defective mechanical components requiring surgery.^5 6^ This is often referred to as an impairment-based perspective.^5^ However, OA is not simply a consequence of irreversible structural damage resulting from loading, age or weight; rather, it is a multifactorial condition in which knee health, and thus pain and disability, arises from the interplay between structural, psychological, social and lifestyle factors.^7^ This holistic view has been emphasised in the participatory-based perspective, which frames people as capable, and supports safe and graded activity.^8–10^ A participatory, person-centred approach, grounded in co-learning, co-created goals and coaching, has been associated with greater engagement in self-care and lifestyle-based interventions and can encourage rehabilitation adherence and physical activity in people with knee OA.^8 10^

Clinicians’ beliefs are often impairment-based, a perspective that has been shown to influence what patients think and do to manage their daily life with knee OA.^5 10^ One way to reduce the impact of clinicians’ biases is to use digital rehabilitation. Adaptations of digital content can be driven by standardised protocols and patient-reported data rather than clinician judgement, making digital rehabilitation a promising setting for research on person-centred care from a participatory-based perspective. In addition, digital rehabilitation for knee OA offers a cost-effective, scalable and non-invasive treatment option,^11 12^ thereby facilitating broader implementation of such approaches.

Changing the perspective from an impairment-based to a participatory-based and therefore modifiable condition can empower people with knee OA and increase their engagement in physical activities.^5 9 10^ Adopting this perspective is therefore an important part of evidence-based guidelines, which recommend rehabilitation including education and exercise as the first-line treatment for knee OA.^9 13^ Despite the negative consequences of an impairment-based perspective being known for a decade, it is still a prevailing perspective among clinicians and patients.^6 10^ However, changing the perspective demands an understanding of the individual’s perspectives, including psychological and contextual factors, such as attitudes and beliefs.^6 9 14^

Prior work has examined knee OA-related beliefs, biomechanical versus biopsychosocial framings and the value of participatory-based communication that grounds information in the patient’s context.^5 6 8 9 15^ Yet little is known about how people with knee OA specifically understand the regulation of physical activity, and how these different ways of understanding shape everyday behaviour. Previous research has addressed related phenomena, such as individuals’ perceptions of physical activity and their conceptions of knee OA.^16 17^ These studies suggest that both physical activity and knee OA may be understood in qualitatively different ways, ranging from narrower, more biomedical interpretations to broader, more meaning-oriented understandings. Such differences in understanding are likely to influence how individuals interpret and act on recommendations. For example, a person who perceives physical activity as harmful or as causing wear and tear may interpret the messages in a rehabilitation programme differently compared with someone who perceives physical activity as revitalising. Exploring these different ways of understanding may inform the development of tailored interventions, individually adapting language, educational content and exercise dosing to increase relevance, reduce fear of movement and better support engagement with self-management and lifestyle care.^10^ Such research will not only illuminate the diversity of these ways of understanding but will also enable the development of strategies to support them.

This study aims to explore how patients in digital rehabilitation for knee OA understand the regulation of physical activity, as a foundation for future tailored adaptations of the digital rehabilitation content. It is the first study in a larger research project exploring whether and how person-centred approaches can improve outcomes in digital knee OA rehabilitation.

## Methods

### Design

This qualitative study used a phenomenographic approach to explore variations in how individuals with knee OA who participate in digital rehabilitation understand the regulation of physical activity. Phenomenography is a qualitative research approach developed to explore the different ways people understand or experience a specific phenomenon.^18^ Rather than focusing on individual traits or behaviours, phenomenography maps the variation in ways of understanding across a group, offering insight into how variations in understandings influence perception and action.^19 20^ Originally developed in educational research to support tailored teaching, the approach has since been applied in healthcare to support the development of communication strategies and interventions.^17 21 22^ This design was therefore considered appropriate given the study’s aim. In the context of digital rehabilitation, where patients are expected to independently interpret and act on health information, understanding such variation in ways of understanding may be particularly valuable. The study is reported in accordance with the Consolidated Criteria for Reorting Qualitative Research (COREQ)^23^ and the Standards for Reporting Qualitative Research (SRQR).^24^ The completed COREQ checklist and SRQR checklist^25^ are provided in online supplemental files 3 and 4.

### Participants and procedure

Participants were recruited from patients enrolled in a digital OA rehabilitation programme, Joint Academy. The programme is driven by a private healthcare provider that works in partnership with regional health authorities. The digital rehabilitation programme includes education and individually tailored exercise activities. Each participant was assigned a physiotherapist who monitored progress throughout the programme and provided ongoing support via telephone and chat. Adults (≥18 years) with clinically diagnosed knee OA enrolled in digital knee OA rehabilitation at Joint Academy during the recruitment period from June to September 2025 and who spoke and understood Swedish were eligible. Participants needed access to a smartphone or tablet and sufficient digital literacy to manage simple applications. Exclusion criteria were serious comorbidities, contraindications to exercise and cognitive or sensory impairments preventing informed consent or participation.

In line with phenomenographic guidance, we recruited 20 participants, which is considered typically sufficient to capture the range of ways of understanding within a group.^26^ We used purposive sampling of sex, pain intensity, body mass index and fear avoidance to maximise conceptual diversity, as these factors are associated with functional decline in knee OA.^27^ Physiotherapists informed eligible patients about the study. Interested individuals were then contacted by the first author, who provided both written and verbal information about the study aims, the researcher’s role and their right to withdraw at any time without any consequences for their treatment. After obtaining informed consent, an interview was scheduled. No prior relationship was established before the interviews. The participants were between 47 and 76 years old (median 65.5), and 15 women and 5 men participated. Descriptive demographics of the participants are presented in table 1.

**Table 1:** Demographics of the participants

Characteristics	Value
Sex, n (women/men)	15/5
Age, years, mean (range)	65 (47–76)
Pain, n (NRS categories: mild/moderate/severe)*	1/17/2
Fear avoidance†, n (yes/no)	4/16
Body mass index kg/m^2^, mean (range)	27.5 (20.1–41.6)

* Pain intensity was assessed using the Numeric Rating Scale (NRS, 0–10). Scores were grouped into three clinical categories: mild pain (1–3), moderate pain (4–7) and severe pain (8–10).

† Fear avoidance describes a tendency to avoid physical activity due to beliefs that activity may cause harm or worsen symptoms. In the present study, fear avoidance was measured using a single self-report item: ‘Do you think that physical activity is harmful for your osteoarthritis?’.

### Data collection

An interview guide (online supplemental file 2) was developed and pilot-tested during the first two interviews, after which minor revisions were made. The semistructured interviews followed Ashworth and Lucas’s^28^ guidelines and included: open-ended questioning; empathic listening to elicit meanings, interpretations and understandings; suspending the interviewer’s own concerns and judgements; and using prompts to clarify and encourage elaboration. To further increase validity, the interviewer repeated the same questions several times, asking for exemplifications and encouraging the participants to explore their understanding of how they understand the regulation of physical activity.

Data were collected through individual digital video interviews conducted via Zoom (Zoom Communications, San Jose, California, USA) by the first author (JS). Supplementary field notes were taken immediately after each interview. Confidentiality was maintained by having only the interviewer and participant present. All interviews were audio-recorded and transcribed verbatim using Amberscript (Amberscript Global B.V., Amsterdam, the Netherlands), a cloud-based speech-to-text transcription service. Transcripts were then checked against the original recordings, word by word, for accuracy. The transcripts were not returned to the participants for comment or correction. All the participants completed the interviews, and none dropped out. 20 interviews were conducted, and they lasted for 14–38 min (mean duration 27 min). No repeat interviews were conducted.

### Data analysis

We employed investigator triangulation within a phenomenographic approach, whereby several researchers were involved in the analytic process. The analysis followed principles described by Larsson and Holmström^26^ and is outlined in box 1. Meaning units were identified, compared and grouped into categories of description, which were iteratively refined to represent qualitatively different ways of understanding the phenomenon. The first author (JS) coded all the interviews, in close collaboration with the second author (IKH). The coding was then repeatedly discussed in meetings with all authors. To enhance credibility, a subset of two transcripts was initially coded to identify meaning units responding to the guiding questions and then audited by all the authors. Outputs were compared and discussed until shared understanding was reached, before the coding framework was applied to the remaining material. For each participant, the first author prepared a brief analytic summary identifying their predominant ways of understanding. Dominance was determined based on the way of understanding that was most consistently expressed throughout the interview and that most strongly structured participants’ descriptions of regulation of physical activity. Preliminary categories of description were constructed by grouping meaning units according to their similarities and differences. The analysis involved actively searching for less-dominant and disconfirming cases, examining category boundaries and iteratively articulating the outcome space and its internal relations. An audit trail of decision points was kept, and illustrative data extracts were provided for each category, consistent with Åkerlind.^29^ Data were organised and managed using tables in Microsoft Word (Microsoft Corp., Redmond, Washington, USA).

Box 1 **Seven stages of analysis**

1. Read the whole text.
2. Read again and mark where the participants gave answers to the main interview questions.
3. In these passages look for what the focus of the participants’ attention is and how she/he describes her/his way of regulating physical activity. Make a preliminary description of each participant’s predominant way of understanding the regulation of physical activity.
4. Group the descriptions into categories based on similarities and differences. Formulate categories of description.
5. Look for non-dominant ways of understanding.
6. Find a structure in the outcome space.
7. Assign a metaphor to each category of description.

### Researcher reflexivity and team perspective

The study was conducted by a multidisciplinary team (with competencies within physiotherapy, nursing, digital health, information design and qualitative methodology including phenomenography) in collaboration with a research partner from the Swedish Rheumatism Association. Interviews were conducted in Swedish by JS, the first author. A male physiotherapist and PhD candidate with 20 years of experience working with patients with long-term pain conditions, he has also used digital rehabilitation tools in clinical practice. In addition, he has extensive experience in conducting clinical interviews. This was the interviewer’s first qualitative research interview study, and to support quality, JS worked under supervision from experienced qualitative researchers and kept a reflexive diary throughout the process. Team discussions were used to identify and manage potential biases.

### Preregistration and protocol deviations

The study was preregistered at Open Science Framework (OSF),^30^ and the study protocol is available as online supplemental file 1. During study development the analytical focus was refined towards a phenomenographic exploration of variation in ways of understanding the regulation of physical activity. Consequently, clinical characteristics, such as fear avoidance, were collected primarily to support purposive sampling and describe the sample, rather than to examine associations between descriptive categories and clinical outcomes. One protocol deviation occurred: four participants followed the digital rehabilitation programme for more than the initially planned 2 weeks period before their interview. The deviation was due to practical reasons and was documented on OSF.

### Patient and public involvement

The study design and research questions were developed in dialogue with a patient research partner with knee OA. The patient research partner reviewed the study design and provided feedback on the interview guide, recruitment strategy and the overall relevance of the study from a patient perspective. She also contributed to the analysis phase by commenting on preliminary findings to enhance the interpretative validity. Participants were not asked to comment on the findings, as phenomenography focuses on variation in ways of understanding rather than stable individual views.

## Results

The analysis identified five qualitatively different ways of understanding the regulation of physical activity: avoidance of physical activity, need for expert guidance, navigating symptoms and daily activity, adaptation of physical activity to remain active and development through physical activity. Hereafter, the descriptive categories are referred to as *Avoidance*, *Need*, *Navigating*, *Adaptation* and *Development*. The five descriptive categories relate to each other through shared dimensions. They represent qualitatively different ways of understanding the regulation of physical activity. These understandings shape what aspects are perceived as important and what actions are perceived as possible, as illustrated in the outcome space (figure 1).

**Figure 1:**
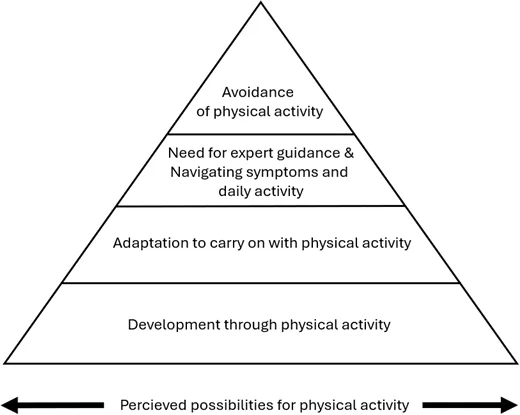
The outcome space illustrates the range of possibilities for engaging in physical activity. It does not represent stages of development, progressions or hierarchy among individuals.

*Avoidance* represents the least complex approach, where pain alone determines regulation of activity. *Need* involves following expert advice and attending to bodily signals, but struggling to interpret them. Physical activity is expected to improve function, but uncertainty about bodily cues persists. *Navigating* involves considering daily life demands and attempting to interpret bodily responses, although often experiencing this as difficult. *Adaptation* recognises multiple ways of being physically active. The intensity, time and form of activity are adapted to be able to continue being physically active. *Adaptation* thereby incorporates more options into regulation. *Development* reflects the most complex approach. With long-term planning that integrates loading, recovery and variation of physical activity, it supports gradual improvement over time.

The descriptive categories do not represent different types of individuals; instead, the same person may express several understandings, although one understanding may appear more dominant in a particular context. See table 2 for an overview of dominant and non-dominant descriptive categories among the participants.

**Table 2:** Overview of dominant (++) and non-dominant (+) ways of understanding the regulation of physical activity

Participant	Avoidance	Need	Navigating	Adaptation	Development
P1			++		+
P2	++		+		
P3				++	+
P4	++		+		
P5				+	++
P6	+		++		
P7			++	+	
P8	+		++		
P9				++	
P10				++	+
P11				+	++
P12	+		+	++	
P13	+		++		
P14				+	++
P15	++		+		
P16	+	++			+
P17				++	
P18				++	+
P19				+	++
P20	+	++			+

Participants may express more than one way of understanding, consistent with a phenomenographic approach.

The descriptive categories are connected through key aspects, often called dimensions of variation, hereafter referred to as dimensions and summarised in table 3. Four dimensions were identified, clarifying how the categories differ. Three of them display a hierarchical structure with increasing complexity. The first dimension concerns perceptions of physical activity, ranging from strain to a means to engage in other activities, to something meaningful in itself, and finally to a way of improving bodily functions within a biopsychosocial frame. The second dimension describes interpretations of pain: from being seen as a signal to stop, to a signal to adapt an activity by modifying its load, intensity, duration or changing the activity, and ultimately to something acceptable when necessary to continue an activity or manage loads required for improvement. The third dimension reflects the perceived purpose of regulating physical activity: from avoiding pain, to managing everyday tasks, to maintaining physical activity, often with the goal of achieving mental comfort or enjoyment, and finally to developing the body through planned strategies involving loading, rest and varied activity. The fourth dimension, perceptions of the body, is non-hierarchical. Participants described the body as subject to wear and tear; difficult to interpret and requiring expert guidance; a limitation hindering meaningful activity; a source of feedback for regulating activity; and as something that can be developed through physical activity.

**Table 3:** Descriptive categories and dimensions of variation

Descriptive categories	**Dimensions of variation**			
	Perceptions of physical activity	Interpretations of pain	Purpose of regulation of physical activity	Perceptions of the body
Avoidance	A strain	A signal of harm	To avoid pain	Subject to wear and tear during physical activity
Need	A means to engage in other activities	A signal to avoid physical activity	To manage day-to-day activity/Improve the body	Difficult to interpret
Navigating	A means to engage in other activities	A signal to avoid physical activity	To manage day-to-day activities	A limitation
Adaptation	Meaningful in itself	A signal to adapt physical activity	To stay physically active	Providing feedback
Development	A means to improve the body	Something that can be accepted	To improve the body	Something that can be developed

### Avoidance of physical activity

The purpose of the regulation of physical activity is, consciously or unconsciously, to avoid discomfort. There is a tendency to plan and to avoid activities in ways that do not trigger or worsen symptoms. The body is understood primarily as a hindrance or limitation to being able to carry out meaningful activities. Physical activity is not experienced as meaningful in itself, but rather serves as a means to engage in other, more highly valued activities, such as shopping. Pain is interpreted as a signal to stop physical activity, and activities are often avoided based on the expectation that they will trigger pain. Regulation of activity is achieved through avoiding activities that are expected to be painful, as explained by one participant:

I think it’s important to make the right movements, so walking far and a lot is not so good for me, because then I get more pain and it strains more than it strengthens the muscles. (P4)

Pain is understood as a sign of injury or degeneration, which shapes the motivation to be physically active. As expressed by P15 when answering the question about the expected long-term effects of physical activity:

Yes, I guess it affects me, so that the more active I am, the less cartilage you see on the screen. (P15)

### Need for expert guidance

How to regulate physical activity is perceived as unclear. Physical activity is understood as beneficial for the body, but only if it is adjusted to the actual circumstances; if the load becomes too great, it may damage the joint. Pain is interpreted as a possible sign of injury, and it is difficult to know how much pain should be accepted during physical activity. There is a perceived need for expert guidance to determine what to do, how to do it and to what extent. Bodily signals are difficult to interpret, making it unclear how much discomfort or pain should be tolerated before an activity should be stopped, as can be seen in the quote of P20:

Then I don’t know, I don’t have the knowledge of, whether it is stupid or whether it is good to keep going when it hurts, for the osteoarthritis, you know? I find that difficult to know. How much pain should you be allowed to have when you walk? (P20)

In this way of understanding, the body appears difficult and unpredictable to interpret. Symptoms may vary from day to day without participants feeling that they understand the cause, which makes it challenging to judge how activity should be regulated. The uncertainty leads to physical activity being limited or stopped when pain arises, as participants do not feel confident in how to interpret bodily signals. As described by P20, participants have a desire for someone with medical competence to determine which activities are appropriate and when it is safe to be more active:

I am afraid that the knee won’t hold up or something will go wrong… If I were to talk, chat or meet someone who says: Yes, well now, now your knee is so good, so go down and work out… More like someone who gets me going and sort of assures me that now I can exercise, you know. (P20)

### Navigating symptoms and daily activity

Regulation of physical activity is guided by everyday activities: e.g. having the responsibility for a dog, needing to shop, playing with grandchildren or carrying out other daily tasks. In this way, physical activity is not in itself meaningful but rather understood as a means to engage in other purposeful activities. Physical activity is something that happens while doing other activities, and it is therefore difficult to articulate how it is regulated, which here is illustrated in the quote of P1:

What is it that determines how active I am? A good question. It could be that. It could. But it’s probably more about what is most practical. Partly when it comes to the dog, and then it can also be that I feel I’m not doing well mentally… But it’s a good question. I actually can’t really answer it. (P1)

The body is understood as a limitation that restricts individuals from doing what they want and from living the life they wish to live. Pain is perceived as a sign of damage and a reason to stop the activity. Pain also affects the motivation to be active, and thoughts about pain can become a hindrance to being physically active, as expressed here:

And the motivation, I mean, you have zero motivation when you’re in pain. I mean, you just become exhausted. It really wears on your mind to be in pain. (P13)

### Adaptation of physical activity to remain active

Physical activity is understood as purposeful in itself and an important part of life that brings happiness and well-being. Inactivity, on the other hand, is associated with a decline in health, particularly mental health. Physical activity can often be maintained even when experiencing symptoms such as pain or discomfort through adaptations in how the activity is performed or by switching to another type of activity or adjusting the load, intensity or duration. As illustrated in the following quote, the body is a source of enjoyment and provides information for regulating physical activity:

You may not be able to do everything, but at least you can do something, and you end up feeling more energetic, happier, and your knee gets better. So there’s really no reason not to. (P3)

Physical activity is often planned to include variation to enable individuals to complete the activity and to remain active in everyday life through purposeful activities that involve physical exertion. Pain is a signal that the activity needs to be adjusted, but at times the pain can be accepted in order to continue with meaningful activities, as described here by P3:

I mean, even if I’m in a lot of pain, I still go (to the training). Because it almost always gets better, and there’s always something I can do. (P3)

The purpose is to be able to do what is meaningful in life. Physical activity is an important aspect of this and is often associated with social activities.

### Development through physical activity

In this category, regulation of physical activity is understood as a way to develop the body to maintain or improve function. The OA is not seen as an obstacle to activity, but rather as a motive to become more physically active, as described by P19:

I can see that, with osteoarthritis in both knees, I really need to make sure that I build up my muscles in such a way that I can keep skiing and doing what I enjoy most in life for many years to come. (P19)

Physical activity is understood as a means to strengthen the musculature and thereby reduce the load on the joint. Increased strength is expected to decrease symptoms in the knee and enable continued participation in activities that are perceived as important in life. Physical activity is often planned and routine-based, and is usually regulated by progressively challenging the body to develop its capacity. Pain is not necessarily understood as a sign of damage, but rather as something that you sometimes need to accept in the process of becoming stronger, as reflected in one participant’s statement:

I mean, it still hurts when I do these movements. But I do them because I know it isn’t dangerous. (P14)

Continuing despite some discomfort may be perceived as part of the progression. At the same time, physical activity is described not only as a means to engage in physical development but also as something that contributes to well-being and a sense of fulfilment, here illustrated by P5:

It’s a feeling of fulfilment, probably because I have a choice. I can do this little extra that I do, and I choose to do it because I want physical well-being. (P5)

## Discussion

### Principal findings

This study identified five qualitatively different ways of understanding the regulation of physical activity among individuals with knee OA participating in digital rehabilitation: Avoidance of physical activity; Need for expert guidance; Navigating symptoms and daily activity; Adaptation of physical activity to remain active and Development through physical activity. Hereafter, the descriptive categories are referred to as *Avoidance*, *Need*, *Navigating*, *Adaptation* and *Development*.

In accordance with phenomenographic theory, these descriptive categories do not represent individual characteristics, beliefs or attitudes. Rather, they describe qualitatively different ways of understanding and experiencing the regulation of physical activity.^31^ Phenomenography assumes that understanding is relational, meaning that it develops through the interaction between the individual and the phenomenon being experienced rather than solely within the individual. Consequently, a person’s understanding of the regulation of physical activity may vary across situations and change over time. At a group level, however, a limited number of qualitatively different ways of understanding a phenomenon can be identified. The descriptive categories therefore represent the variation in how participants understand and make sense of the regulation of physical activity. Variation-theory provides a complementary perspective for understanding how these different ways of understanding may influence the interpretation of information in a rehabilitation programme.^31^

From a variation-theoretical perspective, new experiences and information are interpreted in relation to an individual’s existing understanding of the world. These understandings are shaped by previous experiences and provide a framework through which new information is understood. Consequently, health communication is not received as neutral information but is interpreted in relation to previous experiences and existing ways of understanding physical activity, pain and the regulation of physical activity. The findings show that regulation of physical activity is understood in qualitatively different ways, where differences are not primarily a matter of motivation or level of knowledge, but of how physical activity, pain, the body and the purpose of regulation are perceived as meaningful. These different understandings can be described as different action spaces. Each action space shapes what aspects of a situation are noticed, considered relevant and actively guide action, while other aspects remain in the background of awareness.^31^ For example, ways of understanding characterised by *Avoidance*, *Need* and *Navigating* tend to restrict the range of regulatory alternatives and thereby limit agency, whereas *Adaptation* and *Development* involve a richer differentiation of relevant aspects and support a broader repertoire of regulatory strategies. By making this variation visible, the study contributes to a deeper understanding of why similar health messages may be interpreted differently and lead to different ways of regulating physical activity.

### Comparison with prior work

To our knowledge, no previous studies have specifically explored ways of understanding the regulation of physical activity in individuals with knee OA. However, related aspects, such as perceptions of physical activity and conceptions of knee OA, have been previously described.^16 17^ In line with the present findings, these studies indicate that physical activity and knee OA can be understood in qualitatively different ways, ranging from more narrow interpretations of activity to broader, more meaning-oriented understandings.

The variation identified in the present study can be further interpreted in relation to previously described impairment-oriented and participation-oriented perspectives within knee OA rehabilitation.^10^ The more impairment-oriented ways of understanding identified in this study (eg, *Avoidance*, *Need* and, to some extent, *Navigating*) are characterised by an emphasis on symptoms and perceived bodily constraints, where regulation of physical activity is primarily directed towards controlling or avoiding activity based on bodily signals. In contrast, the participation-oriented ways of understanding (*Adaptation* and *Development*) reflect a shift in focus from symptom control to enabling participation, where regulation is understood as a flexible and context-dependent process aimed at sustaining engagement in everyday life. Although the categories cannot be directly translated into impairment-based or participatory-based perspectives, they help to illustrate how these perspectives may manifest in individuals’ understandings of the regulation of physical activity. As such, the categories contribute to a deeper understanding of how different perspectives are related to different ways of making sense of regulation.

Previous qualitative studies have often emphasised understandings of knee OA as wear and tear or inevitable deterioration.^5 17 32^ The present findings do not negate these understandings but show that, in the current digital rehabilitation context where physical activity is central, regulation of physical activity may also be understood in terms of adaptation and bodily development. This highlights how different contexts can make different aspects of a phenomenon available for understanding.

The current findings are in line with previous research emphasising the need to adapt health communication to the individual. Wallis *et al*^14^ and Bunzli *et al*,^5^ for example, highlight the importance of addressing patients’ beliefs and promoting understandings of the body as active and adaptable, where physical activity is seen as a means to improve function, participation and health through using joints rather than avoiding their use. The present study extends this work by offering a phenomenographic explanation for why such adaptation is necessary. From a variation-theoretical perspective, individuals approach health messages with qualitatively different pre-existing ways of understanding the regulation of physical activity. Effective communication therefore requires more than tailoring information to attitudes or preferences; it involves enabling conceptual change by making critical aspects of regulation evident.^31^ In this sense, communication can be understood as creating conditions for learning, where variation in how regulation is experienced becomes a resource rather than an obstacle.

Previous research exploring how individuals regulate physical activity has predominantly focused on behavioural tendencies and psychological factors, described as activity patterns such as avoidance, persistence and pacing.^33 34^ While these concepts capture how individuals act in daily life, they provide limited insight into how such behaviours are understood and reasoned about. The present findings complement this line of research by illuminating the qualitatively different ways in which individuals understand and make sense of regulation. When interpreted in relation to impairment-oriented and participation-oriented perspectives, a potential link between ways of understanding and activity patterns becomes visible. In the more impairment-oriented ways of understanding, where activity is primarily related to symptoms and perceived risk, regulation may manifest as avoidance or symptom-contingent pacing. In contrast, participation-oriented ways of understanding, characterised by a focus on engagement in valued life situations, may support more flexible and sustainable patterns of activity. This is in line with previous research showing that beliefs about OA influence behaviour and engagement in activity.^35^

Describing ways of understanding the regulation of physical activity may contribute to a deeper understanding of influences on how physical activity is regulated. Previous research has shown that activity patterns are not entirely fixed but may vary across contexts and over time.^34 36^ The present findings complement this research by identifying different ways of understanding the regulation of physical activity. However, as this study was cross-sectional, no conclusions can be drawn regarding whether individuals move between categories over time or whether changes in activity patterns are associated with changes in ways of understanding.

### Strengths and limitations

A strength of the study is the rich variation captured through in-depth interviews, which enabled the identification of distinct ways of understanding the regulation of physical activity. The phenomenographic approach allowed analysis of structural relationships across categories, supported by several dimensions of variation. However, the voluntary participation in a digital rehabilitation programme may have introduced selection bias, as participants may have been more motivated or more positive towards exercise than the broader OA population. Additionally, the data collection through digital interviews may have limited the ability to observe non-verbal cues, although it also increased accessibility and may have facilitated reflection in a familiar environment.

The variable fear avoidance, see table 1, was included solely to describe the sample and to support purposive sampling and was, hence, not used in the phenomenographic analysis. Since it was based on a single item concerning fear of movement rather than a validated measure, no conclusions can be drawn regarding fear avoidance as a psychological construct.

The sample consisted predominantly of women (n=15/20), which may limit the transferability of the findings to men. However, the sample’s gender distribution reflects the gender distribution of the digital rehabilitation programme, from which the participants were recruited. Although the study was not designed to examine gender differences, no specific patterns were observed, with both men and women represented in most categories.

It should be noted that the sample consisted of individuals living in Sweden and who actively chose to participate in a digital exercise-based rehabilitation programme. To minimise the influence of programme content on participants’ understandings, recruitment primarily targeted individuals who had recently enrolled in the programme. Nevertheless, participation in a digital rehabilitation programme may reflect characteristics that differ from those of individuals who are not offered or decline digital rehabilitation, which limits transferability. Furthermore, it remains unclear to what extent the identified ways of understanding are shaped by the digital rehabilitation context or reflect more general ways of understanding the regulation of physical activity among persons with knee OA.

## Conclusion and implications

The findings describe different ways of understanding the regulation of physical activity and how they relate to pain, the body, the purposes attributed to regulating activity and perceptions of physical activity. These ways of understanding were reflected in participants’ descriptions of everyday physical activity and how bodily signals were interpreted in relation to physical activity. Together, these findings suggest that such understandings may influence individuals’ willingness to engage in experiential and exploratory learning through physical activity.

The dimensions of variation identified in this study highlight which subjects clinicians may need to explore to enable more participatory and flexible ways of engaging in activity. This suggests that interventions may benefit from addressing not only behaviour, but also the underlying ways of understanding physical activity, perceptions of pain, perceptions of the body and the purpose of regulating activity. Such an approach could enhance person-centred communication, especially in digital rehabilitation, where adaptations to an individual’s way of understanding may increase the relevance of communication. The categories *Navigating* and *Need* share several characteristics but differ importantly in the reliance on expert advice in the *Need* category. This distinction may be clinically relevant because close adherence to recommendations may mask low trust in one’s own ability to interpret symptoms.

### Future research

Future research should explore how ways of understanding the regulation of physical activity may shift over time and whether they are related to activity patterns and clinical outcomes. It would also be valuable to examine individuals who decline rehabilitation to understand how their ways of understanding the regulation of physical activity differ from those of the current population, consisting of participants in a digital rehabilitation programme. In addition, research should explore how targeted interventions can tailor communication and educational strategies to individual variations in understanding, especially in an evolving digital rehabilitation landscape.

## Supplementary material

10.1136/bmjopen-2026-124247 online supplemental file 1

10.1136/bmjopen-2026-124247 online supplemental file 2

10.1136/bmjopen-2026-124247 online supplemental file 3

10.1136/bmjopen-2026-124247 online supplemental file 4

## Data Availability

Data are not publicly available due to the sensitive nature of the qualitative interview data and the conditions of ethical approval and informed consent. Supporting materials including the inductively developed coding structure, thematic summaries and selected anonymised excerpts may be available from the authors upon reasonable request.
