# Supplementary material for: Ways of understanding the regulation of physical activity among persons with knee osteoarthritis in digital rehabilitation: a phenomenographic study
Source: BMJ Open. 2026 Sep 23;16(9):e124247. doi: 10.1136/bmjopen-2026-124247 (PMC13629973; doi:10.1136/bmjopen-2026-124247)
Supplement: Supplementary file 2 [file bmjopen-2026-124247.reviewer_comments.pdf]

PEER REVIEW HISTORY

BMJ Open publishes all reviews undertaken for accepted manuscripts. Reviewers are asked to complete a checklist review form and are provided with free text boxes to elaborate on their assessment. These free text comments are reproduced below.

ARTICLE DETAILS

Title (Provisional)

Ways of understanding the regulation of physical activity among persons with knee osteoarthritis in digital rehabilitation: A phenomenographic study

Authors

Söderberg, Johan; Holmström, Inger K.; Ehn, Maria; Florin, Ulrika; von Heideken Wågert, Petra

---

VERSION 1 - REVIEW

**Reviewer**	**1**
**Name**	**Thompson, Bronwyn**
**Affiliation**	**University of Otago, Orthopaedic Surgery & Musculoskeletal Medicine**
**Date**	**06-Jul-2026**
**COI**	**None**

This manuscript presents a qualitative, exploratory study using a phenomenographic approach to investigate how patients enrolled in a digital rehabilitation program for knee osteoarthritis (OA) understand the regulation of their physical activity. The research was conducted by a multidisciplinary team from Mälardalen University and Uppsala University, Sweden, with expertise in physiotherapy, nursing, digital health, and qualitative methodology. The senior members of the team, particularly Inger K. Holmström, have an established track record in phenomenographic research. The study identifies five distinct ways of understanding: Avoidance, Need, Navigating, Adaptation, and Development, which range in structural complexity and influence a patient's "action space".

Novelty

The work is highly novel in its specific focus. While extensive literature exists regarding "activity patterns" (behaviours such as pacing or avoidance), the authors correctly identify a gap concerning the underlying conceptual understanding that precedes these behaviours. By shifting the focus from what patients do to how they understand the phenomenon of regulation, the study offers a new framework for understanding why identical clinical advice

results in varied patient responses. To my knowledge, this specific conceptual focus within the context of digital rehabilitation has not been previously published.

Critical Appraisal of Study Design

While the study is rigorously conducted according to COREQ and SRQR guidelines, there are significant design-related tensions that must be addressed:

1. Temporal Claims vs. Cross-Sectional Data: The Discussion interprets changes in activity patterns as "shifts in ways of understanding". However, the study design is cross-sectional, relying on a single 14- to 38-minute interview per participant. Without longitudinal data, the claim that understandings "develop and change over time" or shift toward "increased complexity" is purely speculative and not supported by the evidence provided.

2. Under-utilisation of the Digital Context: The study is situated within "Joint Academy," a digital platform. While the authors acknowledge selection bias, they fail to critically analyse how the digital medium itself (the lack of a physical therapist, the reliance on app-based feedback) might have specifically generated the Need or Navigating categories.

3. Preregistration Discrepancy: The study's OSF registration stated it would provide a basis for investigating the impact of conceptual variation on clinical outcomes (pain, function, quality of life). The current manuscript remains strictly at a descriptive, theoretical level, missing an opportunity to link the identified categories to the clinical demographics (Table 1) already collected.

Major Comments

1. Reframe Speculative Claims: In the Discussion, the authors treat the "shift towards increased complexity" almost as an observed result. You should clarify that these shifts are a hypothesis for future research rather than a finding of the current study, as your data is cross-sectional.

2. Address the "Complexity vs. Hierarchy" Tension: The Results state the categories do not form a hierarchy. However, the Discussion and Figure 1 explicitly use the term "Complexity" and a vertical arrow to rank them. You should provide a clearer theoretical explanation of how a category can be "more complex" without being "hierarchically superior" to avoid clinicians viewing Avoidance as a "lower stage" of personhood.

3. Integrate Digital Influence: The Discussion should explore how the digital rehabilitation environment specifically shaped the relationship between the patient and the phenomenon. For instance, does a digital interface facilitate the Navigating way of understanding by forcing independence, or does it exacerbate the Need for expert guidance category due to the absence of physical validation?

4. Connect Categories to Demographic Profiles: To move beyond abstraction, the authors should briefly discuss if certain clinical profiles (e.g., the 4 participants identified with "fear

avoidance" in Table 1) predominantly aligned with specific categories like Avoidance. This would provide evidentiary weight to your "action space" theory.

Minor Comments

1. Validity of "Fear Avoidance" Metric: The study identifies "fear avoidance" using a single self-report item. This is a limited metric for such a complex psychological construct. Please acknowledge this as a limitation in the relevant section.

2. Sample Gender Imbalance: The sample is skewed toward women (15/20). While common in OA research, the Discussion should briefly note if any gender-specific understandings were observed or if this limits the transferability of the "Development" category.

3. Editorial Polish: The manuscript is generally well-written, but it requires careful editing for minor grammatical flow in the Discussion section to ensure the "variation-theoretical perspective" is accessible to a broad clinical audience.

VERSION 1 - AUTHOR RESPONSE

Reviewer 1

Major Comments

1. Reframe Speculative Claims: In the Discussion, the authors treat the "shift towards increased complexity" almost as an observed result. You should clarify that these shifts are a hypothesis for future research rather than a finding of the current study, as your data is cross-sectional.

We thank the reviewer for this valuable comment. We agree that our cross-sectional design does not permit conclusions regarding shifts in ways of understanding over time. We have therefore revised the manuscript to avoid interpretations that may imply observed developmental change. The discussion now emphasises that the categories represent qualitative variation in ways of understanding identified at one point in time. Furthermore, we have added a recommendation for future research regarding possible shift in ways of understanding and relations to activity patterns and clinical outcomes.

(Page 17, paragraph 3; page 18, paragraph 5; page 19, paragraph 1 and 2)

2. Address the "Complexity vs. Hierarchy" Tension: The Results state the categories do not form a hierarchy. However, the Discussion and Figure 1 explicitly use the term "Complexity" and a vertical arrow to rank them. You should provide a clearer theoretical explanation of how a category can be "more complex" without being "hierarchically superior" to avoid clinicians viewing Avoidance as a "lower stage" of personhood.

We thank the reviewer for this important comment. We agree that the distinction between complexity and hierarchy required further clarification. We have revised the manuscript to explain that the different categories differ in what aspects are perceived

as important and what actions are perceived as possible. We also emphasise that the categories describe qualitatively different ways of understanding the regulation of physical activity, not types of individuals. Accordingly, the categories should not be interpreted as hierarchically superior or inferior, nor as representing a linear progression between ways of understanding. The text and figure legend have been revised to make this distinction clearer.

(Abstract, result & conclusions; page 9, paragraph 3; page 15, paragraph 3; figure 1)

3. Integrate Digital Influence: The Discussion should explore how the digital rehabilitation environment specifically shaped the relationship between the patient and the phenomenon. For instance, does a digital interface facilitate the Navigating way of understanding by forcing independence, or does it exacerbate the Need for expert guidance category due to the absence of physical validation?

We thank the reviewer for this important suggestion. We have added a description of the content in the digital rehabilitation programme under *Participants and procedure* and expanded the *Discussion* to consider the potential influence of the digital rehabilitation context on the findings. The study was designed to recruit participants as early as possible after enrolment in the programme in order to minimise the influence of programme content on participants' understandings. However, we acknowledge that participation in a digital rehabilitation programme may reflect characteristics that distinguish these participants from individuals seeking other forms of care. As our study was not designed to compare digital and non-digital rehabilitation settings, we have refrained from attributing specific categories to the digital context. Instead, we discuss this as an important area for future research.

(Page 5, paragraph 3; page 16, paragraph 3; page 18, paragraph 4)

4. Connect Categories to Demographic Profiles: To move beyond abstraction, the authors should briefly discuss if certain clinical profiles (e.g., the 4 participants identified with "fear avoidance" in Table 1) predominantly aligned with specific categories like Avoidance. This would provide evidentiary weight to your "action space" theory.

We thank the reviewer for this thoughtful suggestion. This was originally within our scope, but during study development the analytical focus was refined towards a phenomenographic exploration. We agree that investigating potential relationships between ways of understanding and clinical characteristics is an important area for future research. However, the present study was conducted using a phenomenographic approach with the aim of describing qualitative variation in ways of understanding the regulation of physical activity rather than examining associations between descriptive categories and participant characteristics. Given the small sample size and the descriptive purpose of the study, we considered the material to be insufficient to

support meaningful conclusions. In addition, the variable referred to as fear avoidance was not assessed using a validated instrument. Instead, a single item concerning fear of movement, routinely collected within the rehabilitation programme was used to support purposive sampling and facilitate variation in participant characteristics. Consequently, no conclusions can be drawn regarding relationships between the identified ways of understanding and fear avoidance as a psychological construct.

To clarify this, we have revised the manuscript and expanded the section *Preregistration and protocol deviations* to explain that clinical characteristics were primarily used to support purposive sampling and sample description rather than to examine associations between categories and clinical outcomes. We have also added a limitation acknowledging that future studies should investigate whether the identified ways of understanding relate to clinical characteristics and outcomes such as pain, function, and quality of life.

(Page 8, paragraph 2; page 18, paragraph 2)

Minor Comments

1. Validity of "Fear Avoidance" Metric: The study identifies "fear avoidance" using a single self-report item. This is a limited metric for such a complex psychological construct. Please acknowledge this as a limitation in the relevant section.

We thank the reviewer for this important observation and agree that fear avoidance is a multifaceted psychological construct that cannot be adequately assessed using a single item. The item concerning fear of movement was included to support purposive sampling and facilitate variation in participant characteristics rather than to measure fear avoidance. We have therefore added a limitation to clarify that fear avoidance was not assessed using a validated instrument and that no conclusions can be drawn regarding fear avoidance as a psychological construct.

(Page 8 paragraph 2; page 18, paragraph 2).

2. Sample Gender Imbalance: The sample is skewed toward women (15/20). While common in OA research, the Discussion should briefly note if any gender-specific understandings were observed or if this limits the transferability of the "Development" category.

We thank the reviewer for this observation. The sample consisted of 15 women and 5 men, reflecting the gender distribution of the digital rehabilitation programme from which participants were recruited. Although the study was not designed to investigate gender differences in ways of understanding, we revisited the descriptive categories and did not observe any clear gender-specific patterns. Men and women were represented across most categories, including the Development category. We have added a brief

discussion of this issue and acknowledge that the predominance of women may influence transferability of the findings to men.

(Page 18, paragraph 3)

3. Editorial Polish: The manuscript is generally well-written, but it requires careful editing for minor grammatical flow in the Discussion section to ensure the "variation-theoretical perspective" is accessible to a broad clinical audience.

We thank the reviewer for this suggestion. We have carefully revised the Discussion section to improve grammatical flow and readability. We have also clarified key variation-theoretical concepts and strengthened the links between the theoretical interpretation and the clinical implications of the findings, making the discussion more accessible to a broad clinical readership.

(Page 15, paragraph 2 & 3; page 17, paragraph 2)

VERSION 2 - REVIEW

**Reviewer**	**1**
**Name**	**Thompson, Bronwyn**
**Affiliation**	**University of Otago, Orthopaedic Surgery & Musculoskeletal Medicine**
**Date**	**07-Sep-2026**
**COI**	

I have no further comments in relation to this revised paper, and recommend it is accepted in the current form.
