# Supplementary material for: Ways of understanding the regulation of physical activity among persons with knee osteoarthritis in digital rehabilitation: a phenomenographic study
Source: BMJ Open. 2026 Sep 23;16(9):e124247. doi: 10.1136/bmjopen-2026-124247 (PMC13629973; doi:10.1136/bmjopen-2026-124247)
Supplement: Supplementary file 3 [file bmjopen-16-9-s001.docx]

**Perceptions of exercise among patients with knee osteoarthritis participating in digital physical therapy**

**This is an update to the original registration. This update was made on Apr 22, 2026.**

Reason for update:

This update documents a clarification of the study objective and a protocol deviation identified prior to analysis, for transparency.

**Study Information**

**Hypotheses**

It is an explorative study aiming to explore and describe different conceptions of physical activity among patients with knee osteoarthritis and therefore it does not have any formal hypotheses.

**Design Plan**

**Study type**

Other

**Blinding**

No blinding is involved in this study.

**Is there any additional blinding in this study?**

*No data*

**Study design**

Qualitative, exploratory phenomenographic interview study.

*No files selected*

**Randomization**

*No data*

**Sampling Plan**

**Existing Data**

Registration prior to creation of data

**Explanation of existing data**

*No data*

**Data collection procedures**

We will recruit participants across Sweden through the private healthcare giver Joint Academy’s digital rehabilitation for knee OA. An initial consecutive sample of 10 patients will be enriched with purposefully selected participants to ensure variation in pain intensity, sex, BMI and fear-avoidance patterns. No financial compensation will be provided. Participants will receive only a small token of appreciation, such as a scratch-off lottery ticket, as a thank you for their time and effort.

*No files selected*

**Sample size**

Previous phenomenographic research has shown that a sample size of twenty interviews is sufficient to capture variations in conceptions within a group. Therefore, this number was chosen for the study.

**Sample size rationale**

*No data*

**Stopping rule**

*No data*

**Variables**

**Manipulated variables**

*No data*

*No files selected*

**Measured variables**

The outcome variable will be conceptions of physical activity, derived through phenomenographically semi-structured interviews.

*No files selected*

**Indices**

*No data*

*No files selected*

**Analysis Plan**

**Statistical models**

Phenomenographic analysis will be used.

*No files selected*

**Transformations**

*No data*

**Inference criteria**

*No data*

**Data exclusion**

*No data*

**Missing data**

*No data*

**Exploratory analysis**

*No data*

**Other**

**Other Updated**

Study updates prior to analysis Clarification of study objective (prior to analysis) The preregistered objective of the study was to explore and describe ways in which patients with knee osteoarthritis undergoing a digital rehabilitation programme conceptualise physical activity. During familiarisation with the interview material and prior to formal analysis, the objective was refined to focus specifically on participants’ understandings of the regulation of physical activity. This refinement represented a clarification of analytical focus within the scope of the original objective and did not constitute a change in study aim, design, or data collection. The refinement was made before analysis commenced and was not informed by study results. Protocol deviation A deviation from the preregistered protocol occurred: four participants followed the digital rehabilitation programme for more than the initially planned two-week period before their interview. The deviation occurred during data collection and was identified prior to analysis. It is not expected to materially affect the results, which are reported at an aggregated group level.

**Metadata**

**Contributors**

[**Johan Söderberg,**](https://osf.io/user/rynw2)

Inger Holmström,

Maria Ehn,

[**Ulrika Florin,**](https://osf.io/user/rcpf9)

[**Petra Heideken Wågert**](https://osf.io/user/2m95e)

**Resource type**

No resource type

**Description**

Background: Knee osteoarthritis (OA) is a progressive, long-term condition whose prevalence is rising in parallel with ageing populations and increasing obesity rates (Global Burden of Disease Study 2021, 2023). The disease often leads to significant suffering, impaired function and increases the risk of lifestyle-related diseases and all-cause mortality (Scheuing et al., 2023). Digital rehabilitation provides a scalable and non-invasive intervention, but patient response is highly variable between individuals (Shah et al., 2021). Evidence-based guidelines do recommend education and exercise as a first-line treatment for knee OA (Conley et al., 2023) but also highlight the necessity to gain a better understanding of factors such as attitudes and beliefs influencing the patient and the need for individualized treatment (Wallis, 2019). It has also been suggested that unhelpful attitudes about OA, can pose barriers to activity (Pulling, 2024). This goes in line with phenomenographic theory which suggests that individuals’ conceptions of physical activity guide their behaviours and experiences (Marton, 1981). Exploring conceptions of physical activity in patients undergoing digital rehabilitation for knee OA may therefore inform the development of more individualised, person-centred treatment strategies. Objective: To explore and describe ways in which patients with knee OA undergoing digital rehabilitation concept physical activity. Methods: We will recruit 20 participants across Sweden through the private healthcare giver Joint Academy’s digital rehabilitation for knee OA. An initial consecutive sample of 10 patients will be enriched with purposefully selected participants to ensure variation in pain intensity, sex, BMI and fear-avoidance patterns. Semi-structured interviews will be conducted via video call or telephone during the first two weeks of the 12-week programme. Interviews will examine how patients perceive and concept physical activity. All sessions will be audio-recorded, transcribed using the Amber transcription service, and analysed with an iterative phenomenographic approach. Descriptive categories will be organized into an outcome space that maps relations among conceptions from simpler to more complex understandings. Expected Outcomes: The study will uncover ways in which patients engaged in digital rehabilitation for knee osteoarthritis conceive and relate to physical activity. We believe that more complex, nuanced conceptions will align with greater behavioural variation, whereas more basic or instrumental views will correspond to narrower spectrum of behaviours. The result will provide a basis for further research investigating the impact of conceptual variation on clinical outcomes (pain, function, quality of life) and for the design of individualized, person‐centred digital interventions.

**Registration Type**

OSF Preregistration

**Registry**

OSF Registries

**Date Created**

May 27, 2025, 11:19 AM

**Date registered**

May 27, 2025, 11:19 AM

**Subjects**

Medicine and Health Sciences

**Tags**

activity patternBeliefsConceptionshabitual activityInterviewPhenomenographyPhysical activityPhysiotherapy

**Affiliated Institutions**

No affiliated institutions

**Funder Names**

No information

**Language**

No language

**Associated project**

[**https://osf.io/k4rbf**](https://osf.io/k4rbf)

**internet archive link**

https://archive.org/details/osf-registrations-n3ubq-v1

**License**

CC-By Attribution-NonCommercial-NoDerivatives 4.0 International

**Registration DOI**

[**10.17605/OSF.IO/N3UBQ**](https://doi.org/10.17605/OSF.IO/N3UBQ)

[Center for Open Science](https://cos.io/)

[Terms of Use](https://osf.io/terms-of-use)|[Privacy Policy](https://osf.io/privacy-policy)|[Status](https://status.cos.io/)|[API](https://developer.osf.io/)|[TOP Guidelines](https://www.cos.io/initiatives/top-guidelines)
