# Supplementary material for: Ways of understanding the regulation of physical activity among persons with knee osteoarthritis in digital rehabilitation: a phenomenographic study
Source: BMJ Open. 2026 Sep 23;16(9):e124247. doi: 10.1136/bmjopen-2026-124247 (PMC13629973; doi:10.1136/bmjopen-2026-124247)
Supplement: Supplementary file 4 [file bmjopen-16-9-s002.docx]

**Interview Guide**

The interview is conducted using a phenomenographic approach, focusing on exploratory questions. The participant is encouraged to provide examples and describe how they perceive the phenomenon of physical activity in their everyday life. The interviewer remains aware of their own pre-understanding and strives to set aside preconceptions and values in order to allow the participant to fully articulate their perceptions of the phenomenon. All information shared during the interview is regarded as equally valuable.

Below is a guide for the research interviews, including example questions:

**Welcome, and thank you for your participation.**
Your experiences and perspectives are very valuable for helping us understand how physical activity is perceived by people with knee osteoarthritis. There are no right or wrong answers, we are interested in your views.

Before we begin, I would like to let you know that you may stop the interview at any time if you wish to take a break or continue on another occasion. The interview will be audio-recorded, but all data will be coded and cannot be linked to your name. If you feel there is anything you would like to change or add after the interview, you are welcome to contact me.

**Interview Questions**

1. Can you tell me about the symptoms that led you to seek care at Joint Academy’s osteoarthritis rehabilitation program?
2. What does physical activity mean to you?
3. What activities in your daily life do you consider to be physically active?
4. How do you experience that physical activity affects your well-being?
5. Can you describe how your level of activity changes from day to day?
6. How do you decide when and how much to be physically active?
7. When you experience pain during activity, how do you manage it?
8. What happens if you take advantage of a day when you feel better and are more physically active than usual?
9. How do you think physical activity affects your knee osteoarthritis in the long term?
10. Is there anything else you think is important to share about your experience of physical activity and knee osteoarthritis that we have not covered?
